# Vaccination in women with cancer: Number 2 - February 2021

**DOI:** 10.1055/s-0041-1726075

**Published:** 2021-02-26

**Authors:** Nilma Antas Neves, Júlio César Teixeira, André Luis Ferreira Santos, Manoel Afonso Guimarães Gonçalves, Fabíola Zoppas Fridman, Cecília Maria Roteli-Martins

**Affiliations:** 1 Universidade Federal da Bahia, Salvador, BA, Brasil.; 2 Universidade Estadual de Campinas, Campinas, SP, Brasil.; 3 Universidade de Taubaté, Taubaté, SP, Brasil.; 4 Pontifícia Universidade Católica do Rio Grande do Sul, Porto Alegre, RS, Brasil.; 5 Hospital Femina Grupo Hospitalar Conceição, Porto Alegre, RS, Brasil.; 6 Faculdade de Medicina do ABC, Santo André, SP, Brasil.

## Key points

- Cancer patients may be immunosuppressed due to their disease of origin or because of anticancer therapies. The degree and duration of immunosuppression varies according to the drug, dose and duration of treatment.
- Immunization of patients with neoplasms must be considered from two points of view: the immunization of the patient herself, as well as the immunization of the family, health professionals and the patient's caregivers.
- The term “immunosuppressed” encompasses different types and degrees of immunosuppression, such as deficiencies in humoral, cellular, complement and phagocytosis immunity. Different impairments of the immune system influence the effectiveness of immunization and the risk of adverse effects.
- Reference Centers for Special Immunobiologicals provide immunobiologicals for routine immunization schedules and special ones for patients in certain conditions.
- Vaccination guidance should result from joint work between the patient's attending physician and Reference Centers for Special Immunobiologicals, as both the vaccination schedule and its respective doses may not follow the usual recommendations.
- Patients who received chemotherapy for the treatment of neoplasms could benefit from booster doses of vaccines, although there is still no defined approach for this situation.

## Recommendations

- Whenever possible, the vaccination schedule should be updated in up to 14 days before the start of antineoplastic therapy. In this situation, the interval between vaccine doses may be shortened.
- After the initiation of therapy that may cause immunosuppression, attenuated vaccines are contraindicated (for example: chickenpox, triple viral, yellow fever, oral polio, oral typhoid and anti-cholera vaccine).
- Patients with neoplasia using immunosuppressive drugs, transplanted solid organs recipients and candidates for transplantation should receive routine vaccines (except attenuated vaccines) and vaccines against pneumococci (depending on the age group); Hib (Haemophilus influenzae type b) for patients up to 19 years; influenza, hepatitis B (regardless of age) and hepatitis A for susceptible individuals.
- Vaccines given during the immunosuppression period need to be repeated after treatment is stopped and the patient is immunocompetent; usually three to six months after the end of treatment.
- Ideal time for vaccination according to immunosuppressive therapies: live virus vaccines should be administered 14 to 30 days before the introduction of immunosuppressive therapy and only three to six months after the end of therapy; may be administered three months after chemotherapy, but at least six months after therapy with anti-B cell antibodies (rituximab). Patients receiving corticosteroids can be vaccinated one month after stopping the drug.
- Children born to mothers who used immunomodulators during the last two trimesters of pregnancy must have the BCG vaccine postponed for 6 to 12 months of life. In this situation, the rotavirus vaccine is not contraindicated.
- Patients who will receive anti-TNF (tumor necrosis factor) should be vaccinated for influenza, pneumococcus, hepatitis B, diphtheria and tetanus. The sooner the vaccination the better.
- Patients susceptible to hepatitis B should be vaccinated before the introduction of rituximab (anti-B cells), because there is a greater risk of complications if this infection occurs.
- In situations of high risk of exposure to the yellow fever virus, the vaccine can be administered to some patients, as long as they are not severely immunosupressed, after medical evaluation. The yellow fever vaccine is not contraindicated for people living with immunocompromised patients. The yellow fever vaccine is contraindicated in immunobiological therapies in which live organisms developed by genetic engineering are used to act on specific targets within the organism.

## Background

Over the past few decades, the number of immunocompromised patients has increased rapidly, although the principles of the state of immunosuppression differ between different categories of patients. Cancer patients may be immunocompromised both because of their neoplastic disease (such as in hematological neoplasms or spinal infiltration by any malignant neoplasm) and by antineoplastic treatment. Generally, immunosuppression in cancer patients is secondary to changes in the cellular and humoral immune response, temporary and resulting from treatment. As this induced depression is intense, in a way, it is predictable, hence, providing immunization before treatments with immunosuppressive potential is a widely used strategy in oncology.

The immunization of cancer patients with a certain degree of immunosuppression that tends to worsen with treatment or by the pathology itself is important for the patient's protection against infections. The family and health professionals who care for these patients also need to be vaccinated against the main infectious agents. If the patient is already immunocompromised, vaccines with live or attenuated agents (e.g. the oral poliovirus vaccine) cannot be used, including for contacts, to avoid the risk of causing a secondary infection in the patient. 1

The vaccination of these women must be considered according to the treatment they will undergo and the people with whom they live. Vaccine guidance should result from a joint work between the patient's attending physician and Reference Centers for Special Immunobiologicals, because the vaccination schedule and vaccine doses may not follow the usual recommendations.

In immunosuppression secondary to chemotherapy, radiotherapy or corticotherapy for cancer treatment, the duration of the immunosuppression condition and the vaccination history are important for the patient's evaluation. The degree of immunosuppression varies according to the type of immunosuppressive drug, dose and duration of treatment.

The great challenge for specialists is to indicate vaccines for patients who will use new therapeutic modalities for the neoplasia, because so far, there are no studies completely determining the safety and effectiveness of vaccines under these conditions.

As the group of cancer patients is very heterogeneous and there are few defining studies on the safety and effectiveness of vaccines in this group, most vaccination recommendations are based on the immune response and vaccine safety in general.

## When should the adult patient with cancer be vaccinated?

Ideally, the patient should update her vaccination schedule and receive the other vaccines indicated for this moment right after the diagnosis of the neoplasia and before starting immunosuppressive therapy. Whenever possible, the vaccination schedule should be updated in up to 14 days before the start of immunosuppressive therapy. In this situation, the vaccination schedule may also be shortened. Whenever possible, we should wait to start the cancer treatment, possibly immunosuppressive; four weeks after application of live vaccines and two weeks after application of inactivated vaccines. 2

The ideal is not to vaccinate during the maximum period of immunosuppression in order to achieve the best immune response and avoid the risk of causing the disease by the vaccine agent. Live vaccines should not be administered during this period. If there is a precise indication due to risky situations, inactivated vaccines can be used during the chemotherapy, radiotherapy or corticotherapy procedure, although reapplication after immunosuppressive treatment will be necessary to ensure an adequate immune response. 3

Three to six months after the end of the immunosuppression condition, the woman can use live, bacterial or viral vaccines, depending on her clinical situation.

Generally, there is no need to revaccinate the patient after chemotherapy or radiotherapy if she has been vaccinated before starting treatment, except patients that received bone marrow transplants, who must be revaccinated. 4 5 6

## Are vaccines safe and effective for patients with cancer?

All inactivated vaccines can be administered to immunocompromised patients, even if the vaccine is of recombinant subunit, fractionated or whole virus, toxoid, polysaccharide or polysaccharide-conjugate. With the exception of the inactivated influenza vaccine, vaccination during chemotherapy or radiation therapy should be avoided, because the immune antibody response should be suboptimal. Patients who were vaccinated within 14 days before the start of treatment or while receiving immunosuppressive therapy should be considered non-immunized and be revaccinated at least three months after the end of immunosuppressive treatment and if immunological competence has been restored. 3

Patients receiving immunoglobulin treatment should not receive live or inactivated vaccines due to low vaccine effectiveness. If they are receiving chemotherapy with anti-B cell antibodies (e.g. rituximab), they should wait at least six months after the end of treatment and then, be vaccinated with inactivated vaccines.

Patients with leukemia, lymphoma or other neoplasms in which disease is in remission, immunocompetence has been reestablished and chemotherapy has ended at least three months earlier, can receive a live virus vaccine. 7

Vaccination against herpes zoster is contraindicated in patients with lymphoma, leukemia, tumors involving the bone marrow and those undergoing chemotherapy. This is a major concern, because the incidence of this disease in patients with altered immunocompetence is significantly high. 8

## What vaccines are recommended for adult cancer patients and their contacts during treatment?

The Advisory Committee on Immunization Practices (ACIP), responsible for vaccination guidance of the Centers for Disease Control and Prevention (CDC), recommends the following specific vaccines for cancer patients: 9

- Pneumococcal vaccines;
- Hib vaccine (Haemophilus influenzae type b): patients under 60 who are undergoing chemotherapy or radiation therapy and have not previously been vaccinated with Hib, and bone marrow transplant patients of any age, regardless of the history of Hib vaccination.

The National Immunization Program (NIP) is responsible for recommending and acting on Brazilian public vaccination, and also recommends other vaccines for cancer patients, shown in Table 1, adapted from the Manual of the Reference Centers for Special Immunobiologicals-NIP-Ministry of Health. 10

Some vaccines outside the vaccination calendar related to the age group are specifically indicated for immunosuppressed patients, such as the pneumococcal, meningococcal and Hib for Haemophilus influenzae.

- There are two pneumococcal vaccines: 13-valent pneumococcal conjugate (VPC13) and 23-valent polysaccharide pneumococcal (VPP23). VPC13 is indicated for children from two months of age and elderly people from 60 years of age, but has a specific dose recommendation for patients of any age with neoplasia. The VPP23 vaccine is also indicated routinely for children from two years old and elderly people from 60 years old and has a specific recommendation of two doses (five-year interval) for patients from two years old with neoplasia. The vaccination schedule should always be started with VPC13, followed by the application of the VPP23 vaccine, respecting the minimum two-month interval in between. After five years, application of the VPP23 should be repeated.
- The meningococcal vaccine is indicated in two doses for immunosuppressed adult patients with a two-month interval. If immunosuppression persists, a booster dose should be applied every five years. Whenever possible, the ACWY meningococcal vaccine should be applied.
- Immunosuppressed patients should also receive two doses of the Haemophilus influenzae type b (Hib) vaccine with a two-month interval between doses.

Vaccines indicated for updating the vaccination schedule are:

- Influenza: prefer the quadrivalent vaccine, as it provides greater coverage of circulating strains. Single annual dose;
- Hepatitis B: if not vaccinated, apply four doses (0-1-2-6 months) in a double dose for the age group. Serology is required after 30 to 60 days. The person is considered vaccinated if anti-HBs is greater than or equal to 10 IU/mL. If serology is negative, the vaccine schedule of four doses with doubled volume should be repeated only once more;
- HPV: the three-dose vaccination schedule is mandatory for immunosuppressed people, regardless of age;
- Triple bacterial: if already vaccinated, booster dose every ten years.

The vaccination of immunocompromised patient contacts is highly recommended. Reference Centers for Special Immunobiologicals provide influenza and chickenpox vaccines to these contacts.

**Chart 1:** Vaccines recommended for patients with neoplasms undergoing chemotherapy, radiotherapy or corticotherapy and for people living with these patients

Vaccine	Patient		Contact
	Before treatment	During treatment	
BCG	No	No	
DTP	Yes	Yes	
OPV	No	No	No
IPV	Yes	Yes	Yes
Hepatitis B	Yes	Yes	
Triple viral	Yes	No	Yes
Chickenpox	Yes	No	Yes, if susceptible
Yellow fever	Yes	No	
Hib	Yes, if <19 years	Yes, if <19 years	
Influenza	Yes	Yes	Yes
Hepatitis A	Yes	Yes	
Meningococcal C (2 doses)	Yes	Yes	
HPV (3 doses)	Yes (9–26 years)	Yes (9–26 years)	
Pneumococcal (according to age) PCV10 / PCV13 / PPSV23	Yes	Yes	

Source: Ministry of Health (2019). 10

## Which vaccines are contraindicated for cancer patients and contact persons during treatment? 2

- Live bacteria vaccines: BCG, adenovirus and Salmonella typhi oral vaccine Ty21a.
- Live virus vaccines: triple viral, oral polio, nasal flu, yellow fever, herpes zoster, rotavirus, chickenpox, dengue and smallpox.

The oral polio vaccine is contraindicated for contacts of immunosuppressed people and should be replaced by the inactivated polio vaccine.

## Are vaccination recommendations different depending on the type of cancer or therapeutic plan?

Immunosuppressive drugs (corticosteroids, immunomodulators, non-biological immunosuppressants, biological immunosuppressants):

The degree of immunosuppression varies according to the drug, dose and duration of treatment. Corticosteroids for oral use are considered immunosuppressants at a dose ≥ 2 mg/kg/day of prednisone (<20 mg/day) or its equivalent. There is no evidence of immunosuppression with the use of topical (skin or eyes), inhalation or intra-articular corticosteroids and there is no contraindication for vaccination in these patients. Patients receiving corticosteroids can be vaccinated one month after stopping them. 5

Immunosuppressant doses of non-biological agents are: 10

- Methotrexate: ≥0.4 mg/kg/week;
- Cyclosporine:> 2.5 mg/kg/day;
- Tacrolimus: 0.1 to 0.2 mg/kg/day;
- Mycophenolate mofetil: 3 g/day
- Azathioprine: 1-3 mg/kg/day;
- Cyclophosphamide: 0.5-2.0 mg/kg/day;
- Leflunomide: 0.25-0.5 mg/kg/day;
- 6-mercaptopurine: 1.5 mg/kg/day.

For biological immunosuppressive agents, any dose is considered immunosuppressive: infliximab (anti-TNFα) and other anti-TNF; rituximab (anti-B cells); abatacept (reduces T cell activation); tocilizumab (anti-interleukin-6); eculizumab (reduces complement activation).

## Transplants in oncology

Various oncological situations require transplants of either solid organs or bone marrow.

**Solid organ transplantation:** the need for immunization of candidates for recipients of solid organ transplants is justified by the immunosuppressive activity of the underlying disease (for example, patients with chronic renal failure and patients with neoplasms) and because they will undergo immunosuppressive therapy after transplantation to avoid rejection of the transplanted organ. Live virus vaccines should not be administered within two months after the end of these drugs, but the herpes zoster vaccine can be administered one month after the end of drugs to prevent rejection. 9

As the transplant may happen at any time, shorter vaccination schedules against hepatitis B can be used. The need to use a double dose should be evaluated according to the underlying clinical situation. Human anti-hepatitis B immunoglobulin (IGHAB) for liver transplant recipients who have AgHbs is regulated by Ordinance No. 86 of February 5, 2002, of the Health Assistance Secretariat and is not responsibility of the Reference Centers for Special Immunobiologicals.

**Bone marrow transplantation (hematopoietic stem cells):** regardless of the type of transplant, the hematopoietic stem cell is responsible for the reconstitution of the immune system of the post-transplant recipient. The post-transplant vaccination recommendation is not different for recipients of autologous, allogeneic or syngeneic transplantation. Studies have shown that hematopoietic stem cell transplant recipients, both allogeneic and autologous, lose protective immunity after transplantation. These individuals must have their vaccination regimen redone after transplantation. 4 5

## Final considerations

Gynecologists have been consolidating themselves as the specialist medical assistants of women with the most opportunity to fully assist them and diagnose gynecological or other organ neoplasms. Women with malignancies will need special care to prevent infectious diseases, including vaccination for various agents, whether to update the vaccination schedule or receive new vaccines indicated because of the neoplasia diagnosis and consequent therapy. As these women must be vaccinated as soon as possible before the start of cancer treatment, the role of gynecologists is key. They will be able to quickly discuss with the oncologist and specialists of Reference Centers for Special Immunobiologicals, speeding up the administration of vaccines to the patient, their relatives and contacts.

National Specialty Commission for Vaccines of the Brazilian Federation of Gynecology and Obstetrics Associations (FEBRASGO)

President:

Cecília Maria Roteli Martins

Vice-President:

Nilma Antas Neves

Secretary:

Susana Cristina Aidé Viviani Fialho

Members:

André Luís Ferreira Santos

Angelina Farias Maia

Fabíola Zoppas Fridman

Giuliane Jesus Lajos

Isabella de Assis Martins Ballalai

Juarez Cunha

Júlio Cesar Teixeira

Manoel Afonso Guimarães Gonçalves

Marcia Marly Winck Yamamoto de Medeiros

Renata Robial

Renato de Ávila Kfouri

Valentino Antonio Magno
